# Targeting the Raft-Associated Akt Signaling in Hepatocellular Carcinoma

**DOI:** 10.1155/2014/836025

**Published:** 2014-08-27

**Authors:** Yuan Liu, Ji-Yun Lv, Jian-Fei Shi, Mei Yang, Shu-Hong Liu, Zhi-Wei Li, Hong-Bo Wang, Shao-Geng Zhang, Zhen-Wen Liu, Jin-Biao Ding, Dong-Ping Xu, Jing-Min Zhao

**Affiliations:** ^1^Department of Pathology and Hepatology, Beijing 302 Hospital, 100 Xi Si Huan Middle Road, Fengtai District, Beijing 100039, China; ^2^First Hepatobiliary Surgery Center, Beijing 302 Hospital, 100 Xi Si Huan Middle Road, Fengtai District, Beijing 100039, China; ^3^Secondary Hepatobiliary Surgery Center, Beijing 302 Hospital, 100 Xi Si Huan Middle Road, Fengtai District, Beijing 100039, China; ^4^Beijing Institute of Infectious Diseases, Beijing 302 Hospital, 100 Xi Si Huan Middle Road, Fengtai District, Beijing 100039, China

## Abstract

Caveolin-1 and flotillin-1 are considered as markers of lipid rafts which can be regarded as sorting platforms for targeted transport of transmembrane proteins and are involved in fundamental cellular events such as signal transduction, cell adhesion, lipid/protein sorting, and human cancer. We addressed caveolin-1 and flotillin-1 expression in 90 human hepatocellular carcinoma (HCC) and adjacent noncancerous tissues (ANT) samples by SDS-PAGE and immunoblotting with specific antibodies. Significant caveolin-1 and flotillin-1 overexpression was found in HCC tissues compared to ANT and was confirmed by immunohistochemistry. Raft-associated Akt signaling pathway components involved in the regulation of cell survival were altered by western blotting in HCC microdomain-enriched subcellular fractions purified from paired HCC and ANT samples. Our results demonstrated that the activity of raft-associated but not total membrane Akt determines its cellular functions. Lipid rafts differ in different types of tissues, which allows for the possibility of tissue-type-specific targeting for cell survival.

## 1. Introduction

Lipid rafts are low-density, detergent-resistant microdomains of plasma membrane (PM) that are enriched in cholesterol and sphingolipids [1–3]. The function of these highly dynamic raft domains as physical platforms for various molecules is involved in a variety of biologic processes by serving as organizing centers for the assembly of signaling molecules into functional complexes [4, 5]. “Caveolae” and “planar lipid rafts” are the two different types of lipid rafts. Caveolae are tube-like invaginations of the PM characterized by specific scaffolding proteins, the caveolins. Planar lipid rafts, on the other hand, are defined as noninvaginated microdomains lacking specific morphological features [3]. Given their crucial involvement in transport, lipid/protein sorting, cell adhesion, and signal transduction, caveolae and planar rafts are suspected to play important roles in various diseases, including cancer. Due to its essential function in variety of biological processes, the protein markers of lipid rafts have been well documented to be involved in initiation and progression of human cancers. Indeed, caveolin-1 and flotillin-1 are considered as markers of caveolae and planar lipid rafts, respectively [3, 6]. Caveolin-1 is a classical hairpin protein that plays a key role in caveolae-mediated endocytosis and transport. Its central roles are involved in caveolar raft formation and the organization of signaling platforms. Caveolin-1 has been found to be highly expressed in some tumors* in vivo* and associated with increased tumor-cell survival, aggressiveness, metastatic potential, and suppression of apoptosis. Flotillin-1 is also an indispensable prerequisite for raft formation in noncaveolar microdomains, which promotes the coassembly of activated and specific glycosylphosphatidylinositol- (GPI-) anchored proteins in the PM microdomains and allows the interaction of signaling molecules. Recently, deregulation of flotillin-1 was found in epithelium-originated cancer, including breast cancer [7], colorectal cancer [8], and esophageal squamous cell carcinoma [9, 10]. These findings suggest that the studies of caveolin-1 and flotillin-1 as potential markers seem to be highly relevant to cancer research.

Hepatocellular carcinoma (HCC) is the fifth most common cancer worldwide, the third leading cause of cancer mortality in the world, and a major form of liver cancer responsible for 90% of the primary malignant liver tumors in adults [11, 12]. Only 30%–40% of patients are amenable to potentially curative therapies, such as surgical resection, because of the advanced stage of disease at the time of diagnosis. Most of the cases (85%) occur in developing countries, with the highest incidence rates Southeast Asia and sub-Saharan Africa [11]. Major risk factors for HCC include environmental factors (such as infection with HBV) and genetic alterations. However, the molecular mechanisms of its development and progression remain largely unknown. Thus, it is critical to understand the etiology and to illustrate the mechanisms underlying HCC initiation and progression and further identify valuable diagnostic and prognostic markers as well as novel therapeutic strategies.

Akt signalling in lipid rafts has only recently been examined as an important oncogenic pathway [12–14]. The binding of growth factors to their receptor tyrosine kinases stimulates the phosphorylation of phosphatidylinositol 3-kinase (PI3K) which localizes in lipid rafts. PI3K converts phosphatidylinositol-4, 5-bisphosphate to phosphotidylinositol-3, 4, 5-trisphosphate (PI (3, 4, 5) P3). Akt translocates to the PM and interacts with PI (3, 4, 5) P3 via its PH domain and is, respectively, phosphorylated at two residues (Thr308 and Ser473) by phosphoinositide-dependent kinase (PDK) and various raft-associated kinases, such as mTORC2. One of the first targets of Akt shown to have direct implications in the regulation of cell survival is a member of the proapoptotic Bcl-2-family and is known as BAD. BAD is attached to lipid rafts in proliferating cells while being associated to mitochondria in apoptotic cells. Once phosphorylated, the phosphoserine residues (Ser136 and Ser112) of BAD form affinity-binding sites for 14-3-3 molecules, thus localizing phosphorylated BAD to the cytosol and effectively neutralizing its proapoptotic activity. Recent studies indicated that raft-associated Akt could be an important determinant of oncogenicity [13, 14]. Studies of small cell lung cancer cells showed that specific PI3K isoforms reside in lipid rafts and that disruption of membrane rafts by Methyl-*β*-cyclodextrin (M*β*CD) inhibits PI3K-mediated Akt activity [15, 16]. Live-cell fluorescence imaging has shown that raft Akt is activated faster and more potently than nonraft Akt, presumably due to compartmentalization of various components of the signaling pathway, including the receptors and Akt itself.

In this study, we investigated the expression of the two most typical membrane microdomain components, caveolin-1 and flotillin-1, in HBV-related HCC and ANT samples and evaluated their clinicopathologic significance in 90 archived HCC samples. Furthermore, we first analyzed the activation status of raft-associated Akt signaling pathway in regulating cell proliferation and survival in HCC.

## 2. Materials and Methods

### 2.1. Patients

For the use of clinical materials for research purposes, prior patients' consents and approval were obtained from Beijing 302 Hospital. All samples were collected and analyzed with prior written informed consents from the patients. HCC was classified according to WHO recommendations also using immunohistochemical techniques; only samples from diagnosed clear HCC were included in the study. Immediately after removal, representative tumor (HCC) and adjacent noncancerous tissues (ANT) of 90 patients were collected by the pathologists and stored in liquid nitrogen until further use between January 2013 and December 2013. The median age of the patients was 49 years (range, 28–78 years old). The tumor histologic grade of all the patients was classified according to the American Joint Committee on Cancer (AJCC) TNM staging system [17]. The clinical and pathological characteristics of the patients are listed in Table 1.

### 2.2. Materials

Antibodies to phosphor-Akt (Ser473), phosphor-Akt (Thr308), Akt (pan), mTOR, BAD, phosphor-BAD (Ser112), and phosphor-BAD (Ser136) were purchased from Cell Signalling Technology, Inc. (Massachusetts, USA). Antibodies to caveolin-1 and flotillin-1 were purchased from Abcam, Inc. (Massachusetts, USA). *β*-Actin antibody, HRP-conjugated anti-rabbit, and anti-mouse IgG were purchased from CWBio, Inc. (Beijing, CN). Protease inhibitor cocktails were obtained from Roche Molecular Biochemicals (Mannheim, DE). BCA protein assay kit and PVDF membranes were purchased from BIO-RAD (Hercules, CA). Masson-Trichrome stain kit and reticulin stain kit were purchased from Yili Fine Chemicals (Beijing, CN).

### 2.3. Lipid Raft Isolation

After hepatectomy, the fresh liver tissue samples obtained were submitted to subcellular fractionation through differential centrifugation. Samples were kept on ice during all the subsequent steps. Briefly, surgical tissues were weighed and minced with scissors. Samples were mixed with tissue lysis buffer (150 mM NaCl, 20 mM Na_2_HPO_4_, 2 mM NaH_2_PO_4_, 20% (v/v) glycerol, and 2 mM sodium orthovanadate with protease inhibitors, pH 7.4) and homogenized 30 times with a tight Dounce homogenizer. Samples were further disrupted by intermittent sonication (six 30 s pulses with a 1 min cooling period between) and then centrifuged at 10 K rpm (Beckman-Coulter Optima L-90K ultracentrifuge with an SW55Ti rotor) for 11 min at 4°C to separate cell debris and nuclear materials. The supernatant was then centrifuged at 32.5 K rpm (SW55Ti rotor) for 90 min at 4°C to pellet the PM. The PM was suspended and solubilised in 3 mL solubilising buffer containing 0.5% v/v Triton X-100 in Mes-buffered saline (MBS: 25 mM Mes, pH 6.5, 0.15 M NaCl), protease inhibitors, and 2 mM sodium orthovanadate for 15 min on ice. Then, 1 mL of solubilised PM was subpackaged and stored in −80°C until use. 2 mL of solubilised PM was further diluted with an equal volume of 80% sucrose in MBS and loaded on the bottom of a 13 mL ultracentrifuge tube overlaid with 4 mL of 30% sucrose/MBS. Finally, 4 mL of a 5% sucrose/MBS solution was added as the top layer of the gradient. The gradient was centrifugated at 31 K rpm (SW41Ti rotor) for 16 h at 4°C to isolate the lipid raft and nonraft compartments. The gradient was then fractionated into 12 fractions [13].

### 2.4. Western Blotting

The protein concentration was measured using BCA protein assay. An equal volume of fraction (10 *μ*L/lane) from each sample was separated on a polyacrylamide gel. For immunoblotting, the proteins were transferred onto PVDF membranes using a wet transfer system (BIO-RAD). The membranes were blocked with 5% nonfat milk (BIO-RAD) for 1 h at room temperature and probed with primary antibodies at 4°C overnight. The membranes were then washed three times with TBST (Tris buffered saline with 0.05% Tween 20) and incubated with HRP-conjugated anti-rabbit or mouse IgG (1 : 5000) for 1 h at room temperature. Signals were detected using enhanced chemiluminescence (Perkin-Elmer Life Sciences, Massachusetts, USA) and band intensities were quantified using Image J software (NIH) [18, 19].

### 2.5. Immunohistochemistry

Tumor liver samples were formalin-fixed and paraffin embedded. 5 *μ*m thick tissue sections were deparaffinaged with xylene and graded alcohol orderly. Then the tissue sections were washed with phosphate buffered saline solution (PBS, pH 7.4) and repaired antigen with citral buffered solution (pH 6.0). Endogenous peroxidase was blocked by incubation for 10 min with 3% hydrogen peroxide in deionized water and blocked with normal goat serum. Sections were then incubated overnight at 4°C with antibodies to caveolin-1 (rabbit, 1 : 200) and flotillin-1 (rabbit, 1 : 200). Antigen-antibody detection was performed with an anti-rabbit peroxidase-conjugated secondary antibody for 1 h at room temperature and DAB colorated and counterstained with haematoxylin. Finally, sections were dehydrated with graded alcohol and mounted [18, 19]. For negative controls, the primary antibody was replaced with normal nonimmune serum. The sections were reviewed and scored independently by two observers.

### 2.6. Special Stains

To assess the cirrhotic status in HCC patients, paraffin sections prepared from HCC samples were stained using Masson-Trichrome method to identify type I collagen and assess fibrosis and reticulin method to identify type III collagen and evaluate lobular architecture and hepatocyte plate thickness. Masson-Trichrome stain and reticulin stain were done as described in the kit manual.

### 2.7. Statistical Analysis

All statistical analyses were performed using the SPSS 11.0 statistical software package and GraphPad Prism 5.0 software. Comparisons between groups for statistical significance were performed with one-way ANOVA, post hoc comparisons being made by using the nonparametric Dunn's multiple comparisons test. *P* < 0.05 in all tests was considered statistically significant.

## 3. Results

### 3.1. Overexpression of Caveolin-1 and Flotillin-1 Proteins in the HCC Samples

**Figure 1:**
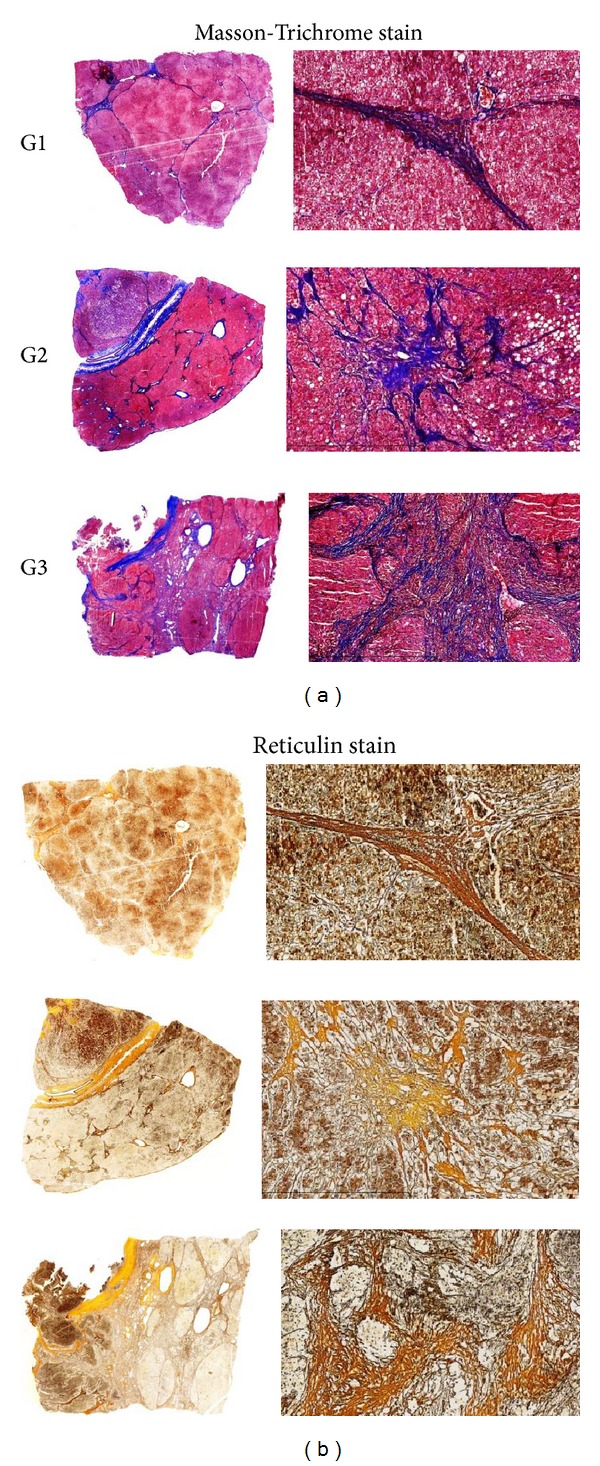
The cirrhotic status in histology. All images are histological finding of cirrhosis but show different feature in thickness of septa and the size of nodules in HCC specimens of different histologic grades. (a) Masson-Trichrome stain, magnified one time and 100 times respectively; (b) Reticulin stain (magnification, ×1 and ×100). G1 (well differentiation) shows mild cirrhosis with thin septa; G2 (moderately differentiation) shows moderate cirrhosis with at least two broad septa; G3 (poorly differentiation) shows severe cirrhosis with at least one very broad septa.

**Figure 2:**
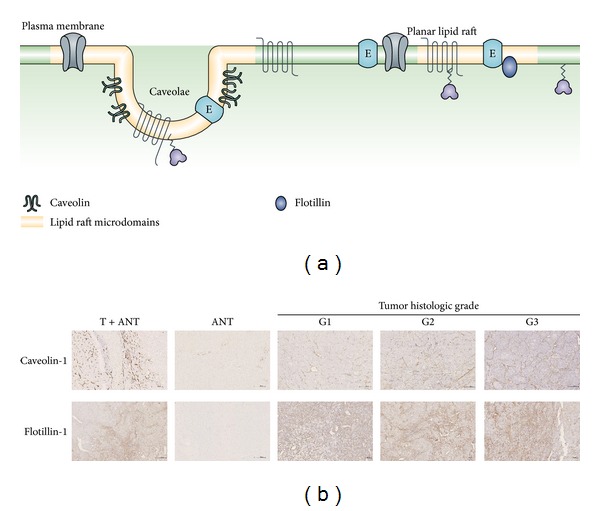
Upregulation of caveolin-1 and flotillin-1 in HCC tissues. (a) Caveolae and planar lipid raft are specialized plasma membrane microdomains that are enriched in cholesterol and sphingolipids and are involved in fundamental cellular events such as signal transduction, cell adhesion, lipid/protein sorting, and human cancer. Caveolin-1 and flotillin-1 are considered as markers of caveolae and planar lipid raft which can recruit signaling molecules into lipid rafts. (b) Representative images from immunohistochemistry analyses of caveolin-1 and flotillin-1 expression in HCC tissues (T) and matched adjacent noncancerous tissues (ANT) and HCC specimens of different histologic grades.

To assess the cirrhotic status in HCC patients, as illustrated by Masson-Trichrome stain and reticulin stain (Figure 1), the liver tissues surgically resected due to HCC exhibited fibrosis with different feature in thickness of septa and the size of nodules in HCC specimens of different histologic grades. To determine the roles of caveolin-1 and flotillin-1 in the clinical progression of HCC, IHC analysis was performed in 90 paraffin-embedded, archived HCC tissue samples, including 30 cases of grade I (G1, well differentiation), 30 cases of grade II (G2, moderately differentiation), and 30 cases of grade III (G3, poorly differentiation) tumors. As shown in Figure 2, high levels of caveolin-1 and flotillin-1 were present in the cancerous tissues of HCC patients. In contrast, caveolin-1 and flotillin-1 were negatively or only weakly detectable in the adjacent noncancerous tissues. In addition, the expression of caveolin-1 and flotillin-1 staining was significantly increased along with the progression of tumor grades I to III. We also noticed that the majority of caveolin-1 staining was membranous in different differentiated HCC samples, whereas the staining of flotillin-1 was strongly both membranous and cytoplasmic.

**Figure 3:**
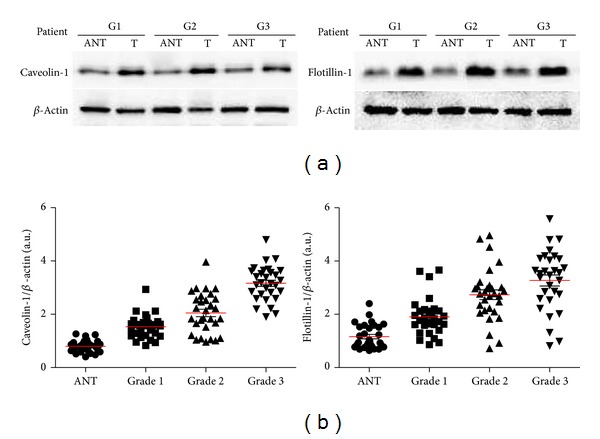
Caveolin-1 and flotillin-1 protein expression in homogenates of HCC and ANT. (a) Western blotting analysis of whole tissue homogenates (25 *μ*g protein/lane) prepared from matched HCC and ANT samples in different histologic grades probed with antibodies to caveolin-1 (left) and flotillin-1 (right) as indicated and simultaneously with antibody to *β*-actin. (b) Densitometric quantification of bands relative to caveolin-1 (left) and flotillin-1 (right) in HCC and ANT tissue homogenates of 30 patients in each group, after SDS-PAGE immunodetection. Band intensity is normalized to *β*-actin.

In order to get confirmation of caveolin-1 and flotillin-1 overexpression in HCC, whole tissue homogenates were prepared from a series of 90 HCC and ANT in different histologic grade. Equal amounts of proteins were used for SDS-PAGE, followed by western blotting and detection with anticaveolin-1 and antiflotillin-1 antibodies and anti-*β*-actin for normalization; in most cases caveolin-1 and flotillin-1 bands resulted more intensely in HCC tissue samples than in autologous ANT (Figure 3(a)). The densitometric analyses of caveolin-1, flotillin-1, and *β*-actin bands for protein loading correction were performed. Quantitative evaluation performed after normalization confirmed the results (Figure 3(b)); overexpression of caveolin-1 and flotillin-1 was recorded in most tumor samples compared to control ones. The differences were statistically significant (*P* < 0.05 both for caveolin-1 and for flotillin-1).

### 3.2. Caveolin-1 and Flotillin-1 Protein Levels in Microdomain-Enriched Fractions

**Figure 4:**
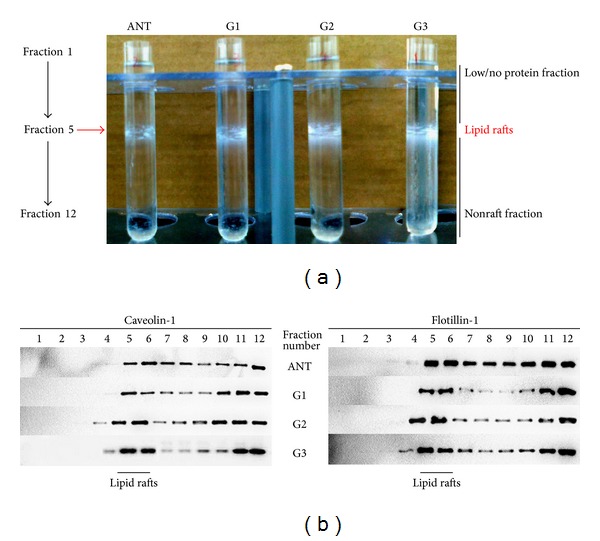
Isolation and identification of lipid rafts in matched HCC and ANT tissues in different histologic grades. (a) Membrane preparations after centrifugation to isolate lipid rafts as described in Section 2. Lipid rafts are mainly in fractions 5 and 6 appearing as an opaque band. Representative image shows an expanded lipid raft collar in HCC samples compared to ANT. (b) Equal volumes of the gradient fractions shown in (a) are analyzed by SDS-PAGE and blotted with caveolin-1 and flotillin-1. As the lipid raft resident proteins, caveolin-1 and flotillin-1 are mainly presented in fractions 5 and 6. All results are representative of at least three independent experiments.

We addressed caveolin-1 and flotillin-1 levels also in detergent-resistant membranes isolated from 10 HCC and paired ANT. In Figure 4(a), lipid rafts were mainly located in fractions 5 and 6 appearing as an opaque band. Representative image showed an expanded lipid raft collar in HCC samples compared to ANT. Analysis of the distribution of lipid rafts along gradient fractions is shown in Figure 4(a), as the lipid raft resident proteins, caveolin-1 and flotillin-1, are mainly presented in fractions 5 and 6 of both tumor and normal tissues (Figure 4(b)). Furthermore, levels of caveolin-1 and flotillin-1 proteins in HCC samples increased in fractions 5 and 6 and decreased in nonraft fractions compared with ANT samples.

### 3.3. Raft-Associated Akt Signaling Involved in Regulating Cell Survival Is Altered in HCC Tissue Samples

**Figure 5:**
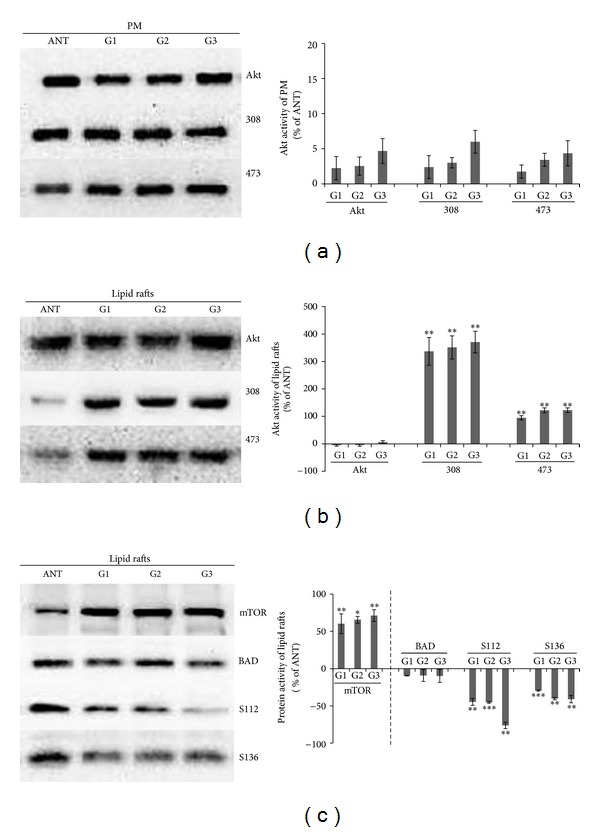
Akt signaling pathway components involved in the regulation of cell survival are altered in lipid rafts of HCC patients. (a) There are no significant changes in the Akt concentration or its phosphorylation state in the plasma membrane of HCC and ANT tissues. (b) The amount of Akt phosphorylation in the lipid rafts significantly increased in HCC compared to ANT tissues. (c) The level of mTOR significantly increased but, respectively, decreased in phosphorylation of BAD in the lipid rafts of HCC tissues. ^∗^ *P* < 0.05, ^∗∗^ *P* < 0.01, and ^∗∗∗^ *P* < 0.001 compared with the ANT,* t*-test (*n* = 3 independent experiments).

We then asked whether the levels of Akt and its phosphorylation status were altered in the PM of HCC and ANT samples. Western blotting of total Akt and its two phosphorylation residues, Thr308 and Ser473, were performed with total membrane proteins.  Figure 5(a) showed that there were no significant changes in Akt or its state of phosphorylation in the PM of HCC and ANT samples.

Interestingly, Akt and its phosphorylation state were totally different in the lipid rafts isolated from the two different types of tissues. As shown in Figure 5(b), although the total level of Akt did not significantly change in the lipid rafts, phosphorylation at Thr308 and Ser473 was significantly increased in HCC samples, indicating that HCC may stimulate raft-associated Akt activity.

To examine whether the contrasting effects of HCC on raft-associated Akt correlate with a difference in the downstream pathway in the lipid rafts, we, respectively, measured the expression of mTOR, BAD, and phosphorylated BAD, which are the downstream targets of Akt. mTOR, a master kinase, plays a critical role in the regulation of tumor cell aggressiveness and cancer metastasis and BAD phosphorylation is an important antiapoptotic mechanism of Akt signaling.  Figure 5(c) showed that mTOR activity significantly increased in HCC samples. In contrast, levels of BAD phosphorylation in HCC samples were significantly reduced, but neither HCC nor ANT significantly altered the total levels of BAD in the lipid rafts. Moreover, the expression of BAD phosphorylation was significantly reduced along with the progression of tumor grades I to III.

## 4. Discussion

In the present study we demonstrate the overexpression of two typical lipid raft resident proteins, namely, caveolin-1 and flotillin-1, in HCC compared to ANT. Caveolin-1 and flotillin-1 expression were analyzed both in total homogenates and in lipid raft fractions prepared from freshly processed human tissues taken from living subjects. We could confirm the upregulation of caveolin-1 and flotillin-1 in HCC, by immunohistochemistry on tissues and by western blotting analysis of the purified microdomain fractions. It has to be underlined that the identification of caveolin-1 and flotillin-1 was achievable only in the purified microdomain fractions, being these proteins characterized by very low abundance in the total homogenates. Overexpression of caveolin-1 and flotillin-1 could increase the number of lipid rafts, whereas knockdown of caveolin-1 and flotillin-1 disrupted lipid raft formation [11, 20]. As the opacity might indicate the presence of a specific form of complex containing insoluble proteins and lipids, an expanded lipid raft collar in HCC samples clearly suggests that the physical state of the proteins can be altered in the lipid rafts.

Several reports indicate that caveolin-1 has a role in cancer development and progression [8, 21, 22]. A strong correlation was found between caveolin-1 expression and the presence of venous invasion of HCC samples (*P* = 0.02) [20]. In fact, in a series of studies* in vivo* researchers found caveolin-1 upregulation in several tumors, such as prostate cancer [23], lung cancer [24], and breast cancer [22]. Moreover, some recent papers report caveolin-1 overexpression in HCC and its correlation with prognostic factors [21]. However, these latest studies were all undertaken using only immunohistochemical evaluation, and in most cases, caveolin-1 overexpression was recorded in the cytoplasm of liver carcinoma cells. Our study, relying on subcellular fractionation, allows a more precise localization. Indeed, no or only weak signal for caveolin-1 was detectable in the cytoplasm fractions of HCC compared to ANT by western blotting. This means that although the presence of minor amounts of intracellular caveolin-1 is confirmed in different cellular models, it may derive from intracellular membrane-bound caveolin-1. Therefore, our data provides the first demonstration of caveolin-1 overexpression in HCC caveolar microdomains. With respect to flotillin-1, much less information is available from the literature than for caveolin-1. In fact, flotillin-1 differential expression has been sporadically reported in pathologic conditions such as breast cancer [7], colorectal cancer [8], and esophageal squamous cell carcinoma [9, 10]. Therefore, our work is the first report of flotillin-1 overexpression in the planar lipid rafts of HCC. Remarkably, a recent study, performed in HCC patients and cell culture models, showed that flotillin-1 high-expression was associated with aggressive characteristics of HCC and suggested the possibility of its use as a prognostic marker in patients with HCC [11]. Therefore, we can further confirm that lipid rafts play an important role in HCC.

The most striking finding of the present investigation was that the raft-associated Akt pathway was activated in HCC patients. Our results showed that the total level of Akt in the lipid rafts and PM did not significantly change, but phosphorylation at Thr308 and Ser473 was significantly increased in HCC samples compared to ANT samples, indicating that raft-associated Akt activity may be stimulated by HCC. As it is possible that HCC and ANT tissues may differ in the respective structures of lipid rafts such as those reflecting the composition of resident proteins and that these differences could be elucidated with lipid raft proteomic analysis. It is interesting that, in the HCC tissues, the expression of raft-associated mTOR was significantly increased, but the amounts of phosphorylated BAD were significantly reduced in the lipid rafts, without changing the total amount of raft-associated BAD. Both mTOR and BAD are the phosphorylating targets of Akt. As there was no significant difference in the total BAD activity, it appears that the ratio of phosphorylated BAD to nonphosphorylated BAD was reduced in the lipid rafts by removing of the former from these structures. These bidirectional effects on mTOR and BAD in HCC tissues were in agreement with the survival, aggressive, metastatic, and antiapoptotic effects of tumor cells. As the significant changes of Akt, mTOR, and BAD along with the progression of tumor grades I to III, it may correlate with the increasing number of lipid rafts. More importantly, the fact that tumor-cell survival solely correlate with Akt activity of lipid rafts instead of nonraft compartments in the PM further emphasizes the notion that only raft-associated Akt determines the functions of its signaling.

In conclusion, we show that only phosphorylation of raft-associated Akt determines the activity and direction of Akt signaling. This underlines the importance of focusing on membrane microdomains instead of the global cellular membrane when the functions of signaling proteins are studied. Our results are the first to clearly demonstrate that tissues can be differentially targeted according to differences in the structures of their membrane microdomains.

## Figures and Tables

**Table 1:** Clinical and pathological characteristics of patients enrolled in the study.

	HCC	Histologic grade (G)		
		G1	G2	G3
Case, *n*	90	30	30	30
Age (years)	49.4 ± 9.7	53.7 ± 10.2	47.7 ± 7.3	46.8 ± 10.2
Sex				
Female	17	3	4	10
Male	73	27	26	20
HBV status				
Positive	90	30	30	30
Negative	0	0	0	0
Liver cirrhosis				
Positive	79	23	26	30
Negative	11	7	4	0
